# Genomic Architecture of Yield Performance of an Elite Rice Hybrid Revealed by its Derived Recombinant Inbred Line and Their Backcross Hybrid Populations

**DOI:** 10.1186/s12284-022-00595-z

**Published:** 2022-10-01

**Authors:** Fan Zhang, Conghe Zhang, Xiuqin Zhao, Shuangbing Zhu, Kai Chen, Guixiang Zhou, Zhichao Wu, Min Li, Tianqing Zheng, Wensheng Wang, Zhi Yan, Qinyong Fei, Zhikang Li, Jinjie Chen, Jianlong Xu

**Affiliations:** 1 Institute of Crop Sciences/National Key Facility for Crop Gene Resources and Genetic Improvement, Chinese Academy of Agricultural Sciences, Beijing, 100081 China; 2 Winall Hi-Tech Seed Co., Ltd., Hefei, 230088 Anhui China; 3 College of Agronomy, Anhui Agricultural University, Hefei, 230036 Anhui China; 4 Shenzhen Branch, Guangdong Laboratory for Lingnan Modern Agriculture, Agricultural Genomics Institute at Shenzhen, Chinese Academy of Agricultural Sciences, Shenzhen, Guangzhou, 518120 China

**Keywords:** Heterosis, Whole-genome sequencing, Haplotype-based mapping, Genomics, Hybrid rice

## Abstract

**Background:**

Since its development and wide adoption in China, hybrid rice has reached the yield plateau for more than three decades. To understand the genetic basis of heterosis in rice and accelerate hybrid rice breeding, the yield performances of the elite rice hybrid, Quan-you-si-miao (QYSM) were genetically dissected by whole-genome sequencing, large-scale phenotyping of 1061 recombined inbred lines (RILs) and 1061 backcross F_1_ (BCF_1_) hybrids derived from QYSM’s parents across three environments and gene-based analyses.

**Results:**

Genome-wide scanning of 13,847 segregating genes between the parents and linkage mapping based on 855 bins across the rice genome and phenotyping experiments across three environments resulted in identification of large numbers of genes, 639 main-effect QTLs (M-QTLs) and 2736 epistatic QTLs with significant additive or heterotic effects on the trait performances of the combined population consisting of RILs and BCF_1_ hybrids, most of which were environment-specific. The 324 M-QTLs affecting yield components included 32.7% additive QTLs, 38.0% over-dominant or dominant ones with strong and positive effects and 29.3% under-dominant or incomplete recessive ones with significant negative heterotic effects.

63.6% of 1403 genes with allelic introgression from subspecies *japonica*/*Geng* in the parents of QYSM may have contributed significantly to the enhanced yield performance of QYSM.

**Conclusions:**

The parents of QYSM and related rice hybrids in China carry disproportionally more additive and under-dominant genes/QTLs affecting yield traits. Further focus in *indica*/*Xian* rice breeding should shift back to improving inbred varieties, while breaking yield ceiling of *Xian* hybrids can be achieved by one or combinations of the three strategies: (1) by pyramiding favorable alleles of additive genes, (2) by eliminating or minimizing under-dominant loci, and (3) by pyramiding overdominant/dominant genes polymorphic, particularly those underlying inter-subspecific heterosis.

**Supplementary Information:**

The online version contains supplementary material available at 10.1186/s12284-022-00595-z.

## Background

Food security is one of the greatest challenges of the world’s rapid population growth, and only through sustainable food production can this problem be solved. Commercial crop hybrids have favorable characteristics, such as increased yield, biomass, vigorous vegetative growth and improved tolerances/resistances to biotic and abiotic stresses (Fu et al. 2015). The much enhanced yields of hybrids have been utilized in numerous cereal crops and vegetables (Fujimoto et al. 2018). Exploiting heterosis by developing superior hybrid cultivars plays a key role in increasing crop productivity (Zhou et al. 2012). Heterosis, also known as hybrid vigor, refers to the superior phenotypic performances of hybrids over their parents (Birchler et al. 2010). It is a complex biological phenomenon affected by many factors, and its genetic basis has not been explained accurately. Several major hypotheses for heterosis have been proposed as its genetic basis, including dominance complementation, overdominance and epistasis (Hochholdinger and Hoecker 2007; Goff and Zhang 2013).

Rice (*Oryza sativa* L.) is the staple food of more than half of the world’s population (Tao et al. 2016). Rice productivity has been more than doubled since the Green Revolution in the late 1950s. Unlike outcrossing plant species which tend to show high levels of heterosis, self-pollinated plant species are theoretically expected to show relatively low levels of heterosis. However, the presence of significant levels of heterosis for yield traits was reported in many cases in rice (Jones 1926; Li et al. 2001; Cheng et al. 2007; Wu 2009; Bagheri and Jelodar 2010). In fact, a second leap in rice productivity of China could largely be attributed to the development and wide adoption of hybrid rice in China since the late 1970s when the three-line cytoplasmic male sterility (CMS) became established in rice. Since then, the planting area of ​​hybrid rice has eventually reached over 50% of the total rice area in China until today. Also, increased breeding efforts have been taken to exploit the well-known inter-subspecific heterosis by inter-subspecific introgression in many hybrid rice breeding programs in China. Nevertheless, the productivity of hybrid rice in China has improved slowly since the 1990s despite tremendous breeding efforts.

The slow genetic gain in hybrid rice breeding of China has stimulated considerable efforts in dissecting the genetic basis of heterosis, particularly since the advancement of molecular markers and genome sequencing technology. To date, two general strategies have been used to dissect the genetic basis of heterosis in rice using DNA markers. The first one is based on classical genetic designs of using various types of segregating populations such as F_2_, backcross F_1_ (BCF_1_)_,_ or testcross F_1_ populations derived from distantly related parents or parents of well-known hybrid cultivars (Li et al. 1997, 2001; Yu et al. 1997; Hua et al. 2002; Mei et al. 2003; Wu 2009; Bagheri and Jelodar 2010; Chen et al. 2011; Tao et al. 2016; Xiang et al. 2016). Two major advantages of this strategy are (1) genetic parameters of both additive and non-additive effects of quantitative trait loci (QTLs) as well as epistasis can be more accurately estimated; and (2) these populations are commonly used in hybrid breeding and thus results should be more meaningful to breeders. Two general conclusions were reached regarding the genetic basis of heterosis in rice: (1) large numbers of QTLs are involved in determining the performances and heterosis of rice yield traits; and (2) QTL dominance or overdominance effects and epistasis are all important on yield-related traits (Xin et al. 2011; Bian et al. 2010; Luo et al. 2012; Zhu et al. 2016; Shen et al. 2014, 2022; Li et al. 2001; Wu 2009; Bagheri and Jelodar 2010; Xiong et al. 2021; Ouyang et al. 2022). The second strategy is applying genome-wide association study (GWAS) to dissecting heterosis in random or multiple mapping populations (Huang et al. 2016). Using 1,654,030 single nucleotide polymorphisms (SNPs) of 1495 hybrids and their parental lines of rice, Huang et al. (Huang et al. 2015) reported that heterosis involves superior alleles at large numbers of loci of partial dominance effects. Using a partial diallel design of ~ 500 F_1_ hybrids from crosses between 14 male sterile lines and 39 restorer lines and 50 K-SNP chips for genotyping performed GWAS, Zhen et al. (Zhen et al. 2017) found that the superior alleles at many QTLs are the key contributor to yield heterosis of the common two-line rice hybrids of China. The major advantage of this strategy is to be able to gain insights into the genetic architecture of heterosis for yield traits in a diverse set of rice hybrids (Li et al. 2016; Zhen et al. 2017), though the high perseverance of genetic and field data was required to detect significant QTLs in the rice genome associated with hybrid performances (Latha et al. 2013; Xu et al. 2014; Lin et al. 2020). Unfortunately, it remains unclear how to apply results from the limited theoretical studies to break the yield ceiling of hybrid rice and achieve accelerated genetic gains in hybrid breeding, given the complexity of the genetic systems involved in rice heterosis.

We report here an effort to dissect the genetic basis of the heterosis of a superior hybrid, Quan-you-si-miao (QYSM), and general combining ability (GCA) of its parents, Quan9311A (Q9311A) and Wu-shan-si-miao (WSSM). QYSM is one of the predominant three-line *Xian* (*indica*) hybrid cultivars widely grown in whole south China rice region covering the up, middle and low areas of Yangtze River and southern part. Its parents, Q9311A and WSSM, have many desirable traits such as ideal plant architecture associated with high yield potential and wide adaptation, and now become the most widely used three-line sterile line and elite restorer line for more than 54 two-line and three-line hybrids (https://www.ricedata.cn) that are being widely grown in the whole south China rice region covering the up, middle and low areas of Yangtze River and southern part (Li et al. 2016). By deep analyses of 13,847 segregating genes from Next-generation Sequencing and phenotypic data from replicated phenotyping of combined population consisting of 1061 recombined inbred lines (RILs) derived from the cross Q9311B × WSSM and 1061 BCF_1_ hybrids (the Q9311A × RILs) across three environments, we were able to identify large numbers of QTLs/genes of significant additive and non-additive effects on 12 rice yield-related traits. Our results provide insights into the genetic bases underlying the trait performances and heterosis in the elite rice hybrid and provide useful information to further improve the hybrid through more accurate manipulation of the identified QTLs/genes.

## Results

### Phenotypic Performance of the RILs and Heterosis of Their BCF_1_ Hybrids

**Fig. 1:**
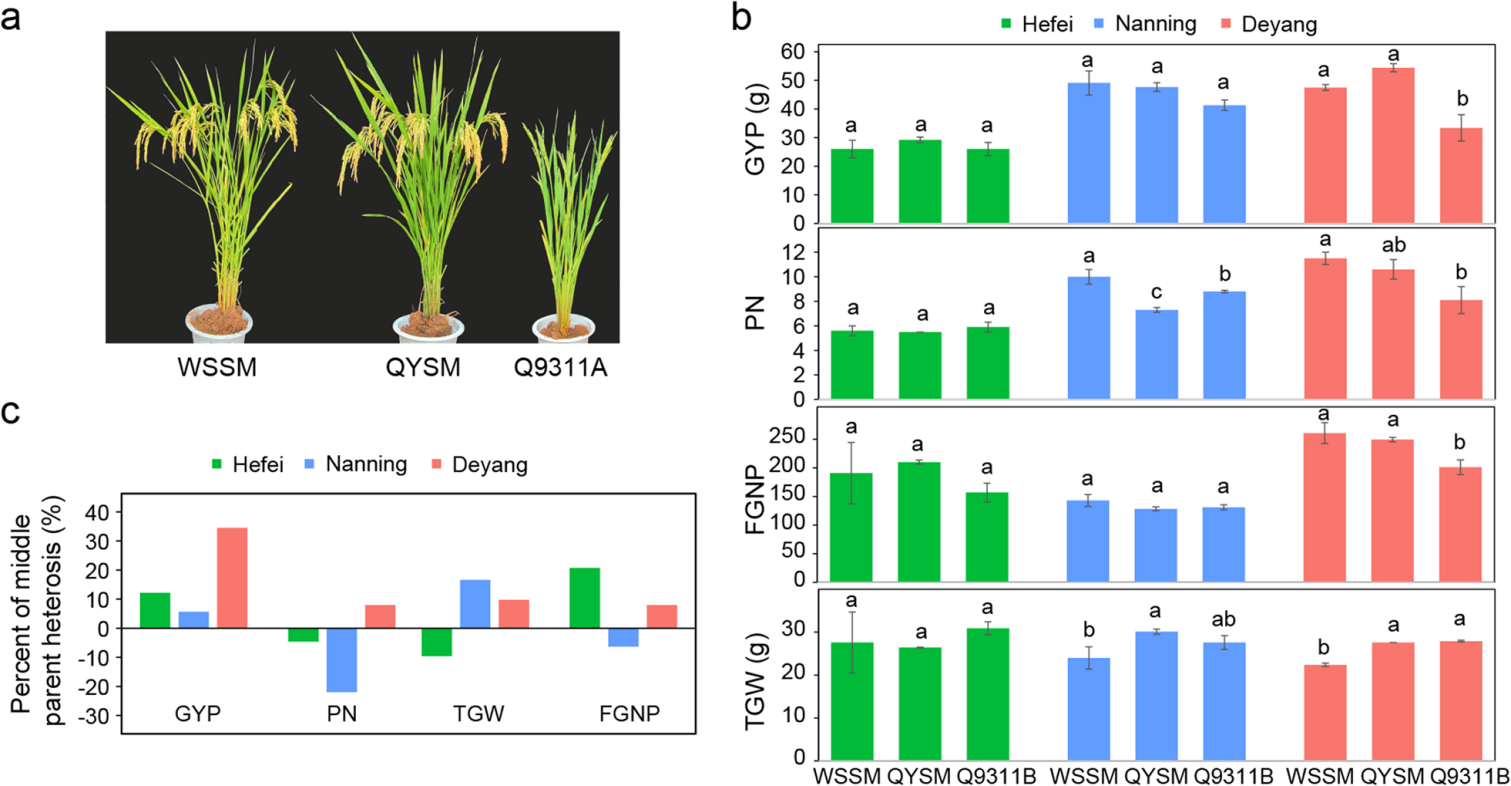
Phenotypic differences and heterosis between the elite rice hybrid, Quan-you-si-miao (QYSM) and its parents. **a** Whole-plant morphology of QYSM and its parental lines Quan9311A (Q9311A) and Wu-shan-si-miao (WSSM). **b** Performance of yield-related traits in QYSM and its two parents in three environments. Different letters above the bars represent significantly differences at *P* < 0.05 based on Duncan’s multiple range test. **c** The middle parent heterosis of yield and yield component traits. GYP, grain yield per plant; PN, panicle number per plant; TGW, 1000-grain weight; FGNP, filled grain number per plant

Figure 1 and Additional file 1: Table S1 show the mean trait values and *H*_MP_ of the hybrid, QYSM, its parents, Q9311A and WSSM, in the three environments, HF, NN and DY. WSSM showed significantly higher values than Q9311B for all traits except for TGW, FLW and SF in at least one environment. The hybrid QYSM showed the same trait values as WSSM for most traits except with significantly lower PN, greater TGW, lower SF, wider FLW and longer PL. QYSM showed variable levels of *H*_MP_ for GYP in the three environments (12.2% in HF, 5.6% in NN, and 34.5% in DY), and so for PN, FGNP and TGW. FGNP was the major contributor to the GYP heterosis of QYSM in HF; TGW was primarily responsible for the low level of GYP heterosis of QYSM in NN, while the high level of GYP heterosis of QYSM in DY was attributed to high *H*_MP_ of all three yield components, PN, FGNP and TGW. This result indicated that the observed yields of QYSM were achieved through varied levels of heterosis of different yield components across different environments. ANOVA indicated that the trait expression of the parents and hybrid QYSM was strongly affected by the three environments, particularly for HD (*R*^2^ = 91.6%), SF (*R*^2^ = 86.8%), FGNP (*R*^2^ = 74.4%), PN (*R*^2^ = 72.9%) and TGNP (61.5%), while significant genotype (G) × environment (E) interactions were observed for all traits, except for HD, FGNP, and SF, though the G × E effects were relatively small compared with the main effects of different genotypes or environments (Additional file 2: Table S2).

The summary statistics of the trait values and *H*_MP_ of the 1061 RILs and their BCF_1_s (Q9311A × RILs) in HF, NN and DY are given in Additional file 3: Fig. S1. The RILs and BCF_1_s showed a continuous and wide distribution for all 12 traits, indicating complex genetic mechanisms underlying the trait performances and their heterosis. ANOVA indicated that the variation among different genotypes (RILs and BCF_1_ hybrids), among different environments, and G × E interactions were all highly significant for all measured traits, and on average, explained 33.0% (30.1%), 29.9% (32.5%) and 22.6% (21.4%) of the total phenotypic variation of the RIL (BCF_1_) populations (Additional file 2: Table S2). Relatively speaking, the among genotype variation was more important for TGW, PL, flag leaf length (FLL), flag leaf area (FLA), SF, FLW and plant height (PH) in the RILs and for SF, FGNP, FLL, PL, GYP, TGW and FLA in the BCF_1_s. Comparatively, HD, GYP, FGNP, TGNP, PN and PH in both the RILs and BCF_1_s were influenced more by the environments. The G × E interactions contributed more evenly to all traits except for HD. Significant and highly positive correlations between PH and GYP and between FGNP and GYP were observed in the BCF_1_s in the three environments, and so were roughly no correlations between PN and GYP in HF and DY. The correlations between TGW and GYP and between HD and GYP were obviously affected by the environments. Linear regression analysis showed that GYP in the BCF_1_s was primarily determined by FGNP (0.616 for HF, 0.249 for NN and 0.558 for DY), as compared with PN (0.003 for HF, 0.039 for NN and 0 for DY) and TGW (0.026 for HF, 0 for NN and 0.042 for DY).

The average *H*_MP_ of the BCF_1_ hybrids was negative for GYP, TGW, PN and FGNP in HF, positive for GYP, TGW, PN and FGNP in DY (Additional file 1: Table S1). In NN, it was zero for GYP, positive for TGW and negative for PN and FGNP. Individual BCF_1_ hybrids showed considerable variation in their *H*_MP_ values for all measured traits, ranging from highly significant negative heterosis to high significantly positive heterosis. When compared with the check hybrid QYSM (standard heterosis), most BCF_1_ hybrids showed significantly lower GYP, only ten, eight, and one of them showed significantly higher GYP than QYSM in HF, NN, and DY, respectively. The ten superior BCF_1_ hybrids in HF had a mean *H*_MP_ of 38.4% for GYP, − 9.0% for PN, 21.0% for FGNP, and 1.5% (not significant) for TGW. The eight superior BCF_1_s in NN showed a mean *H*_MP_ of 43.1% for GYP, − 9.1% for PN, 2.4% (not significant) for FGNP, and 16.1% for TGW. The only superior BCF_1_ hybrid in DY had a *H*_MP_ of 72.5% for GYP, 30.3% for PN, 2.0% (not significant) for FGNP, 7.2% for TGW, and in the BCF_1_ with significantly higher GYP in DY. It appeared that the check hybrid, QYSM has approached the limit in yield potential of the BCF_1_s. Thus, further improving the yield potential of QYSM by conventional breeding would be difficult without a full understanding of the genetic basis of its yield performance in different environments.

### Identification of QTLs of Different Gene Actions

**Fig. 2:**
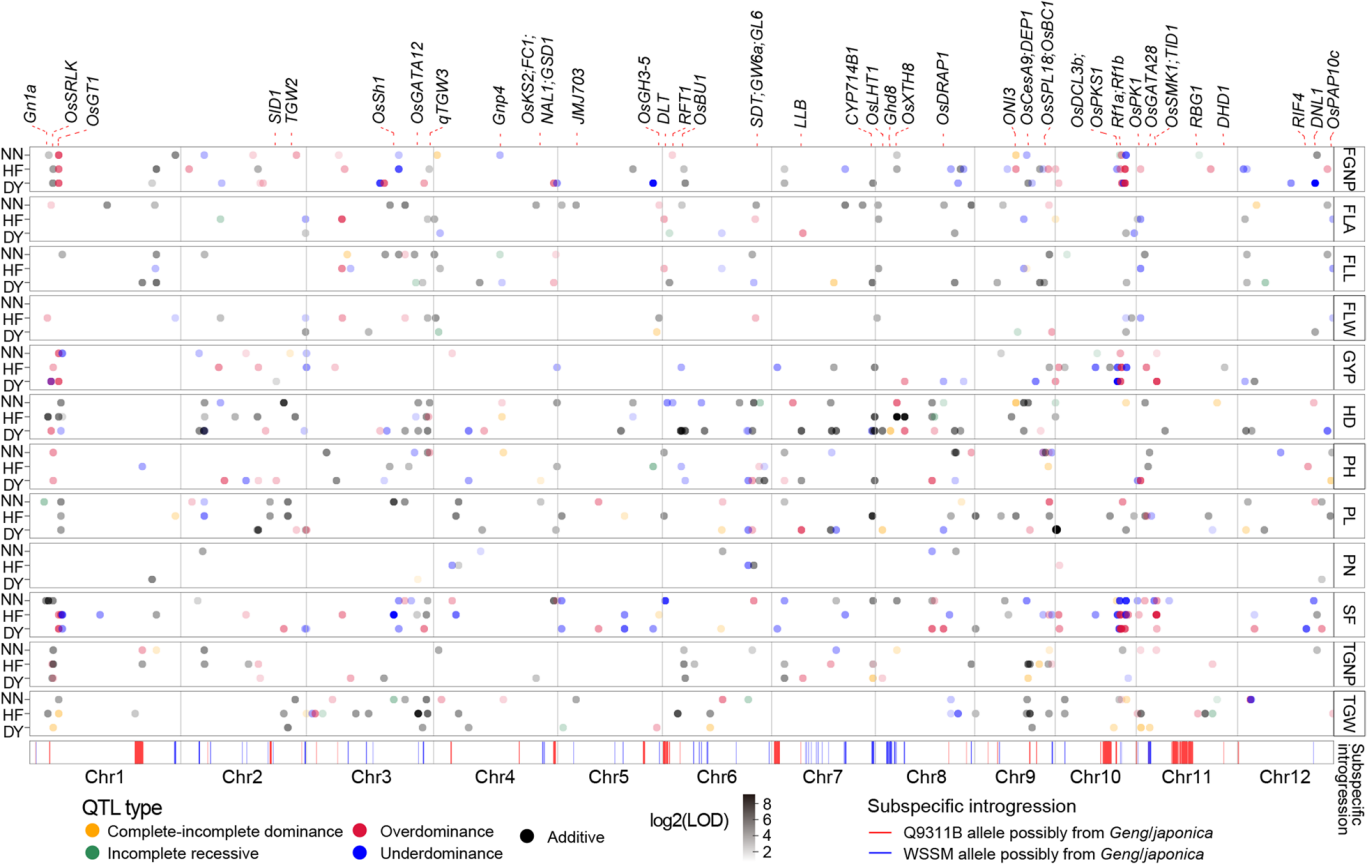
Genomic distribution of 639 main-effect QTLs affecting 12 rice yield related traits identified in the combined (RIL + BCF_1_) population of the Q9311/WSSM cross across three environments (Hefei [HF], Nanning [NN] and Deyang [DY]). On the top are 45 cloned rice genes of known effects on related traits and the bottom are 12 rice chromosomes. The bottom tile track highlights the distribution of genes with *japonica*/*Geng* allelic introgression in the parents of QYSM

**Fig. 3:**
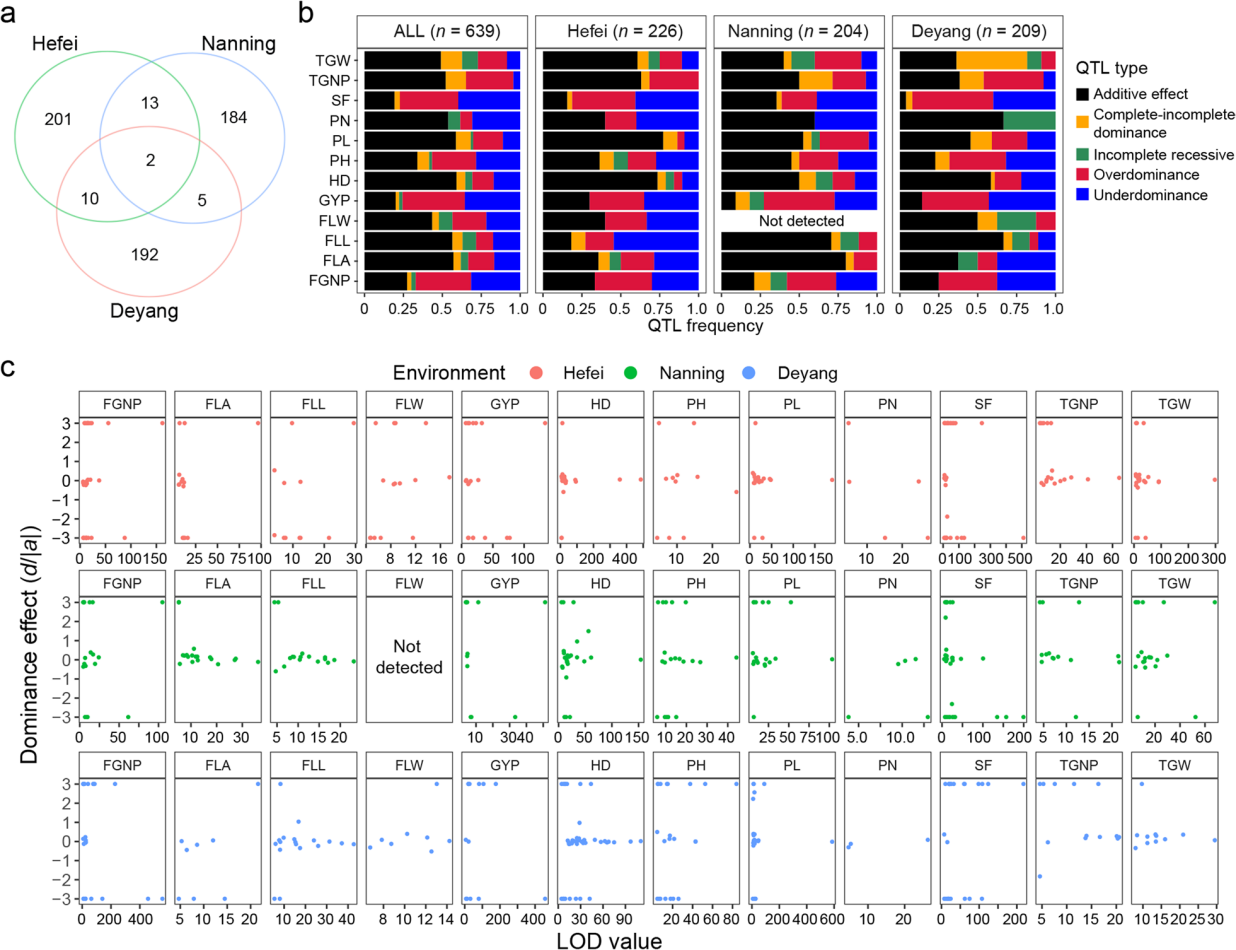
Comparison of the main-effect QTLs showing different gene actions affecting yield traits detected in combined population across three environments. **a** Venn diagram showing the numbers of co-localized QTL among three environments. **b** Frequencies of five gene actions of main-effect QTLs for 12 traits in three environments. **c** Plots of the dominance effects (*d*/|*a*| values) and their LOD values of the QTLs for 12 traits in Hefei, Nanning and Deyang. The QTLs with *d*/|*a*| values of > 3 or < –3 are plotted to be = 3 or = –3 for display purposes

To identify QTLs affecting the measured traits and their gene actions, a high-density linkage map consisting of 855 bins was constructed for the RIL population based on the genotypes at 13,847 segregating genes using the ICIMapping (Additional file 4: Fig. S2a). The total length of the genetic map is 2268 cM and the allelic segregation ratio of these bins fit approximately the Mendelian separation ratio of ~ 50% (Additional file 4: Fig. S2b). The genotypes at all segregating genes (and bins) for each BCF_1_ hybrid were deduced according to the genotypes of the male parental RILs and female parent Q9311A (with the same genotype as Q9311B). We merged the RILs with BCF_1_s as a single combined mapping population with frequencies approximately equivalent to a very large BC_1_F_2_ population for mapping heterotic loci. Based on the bin map constructed, QTL mapping was performed separately for the RIL, BCF_1_, and combined populations using the inclusive composite interval mapping method. The mapping results using the combined population (Fig. 2) were similar to the results using the RIL and BCF_1_ populations (Additional file 5: Fig. S3). A total of 639 M-QTLs were identified for 12 traits in the combined population across the three environments (Additional file 6: Table S3). These included 45 M-QTLs affecting GYP (20 in HF, 11 in NN and 14 in DY), 73 M-QTLs affecting FGNP (30 in HF, 19 in NN and 24 in DY), 13 M-QTLs affecting PN (5 in HF, 5 in NN and 3 in DY), 59 M-QTLs affecting TGW (28 in HF, 20 in NN and 11 in DY), 46 M-QTLs affecting TGNP (19 in HF, 14 in NN and 13 in DY), 88 M-QTLs affecting SF (32 in HF, 31 in NN and 25 in DY), 42 M-QTLs affecting FLA (14 in HF, 20 in NN and 8 in DY), 23 M-QTLs affecting FLW (15 in HF and 8 in DY), 46 M-QTLs affecting FLL (11 in HF, 17 in NN and 18 in DY), 53 M-QTLs affecting PH (11 in HF, 20 in NN and 22 in DY), 63 M-QTLs affecting PL (22 in HF, 19 in NN and 22 in DY), and 88 M-QTLs affecting HD (19 in HF, 28 in NN and 41 in DY), respectively. The detected M-QTLs were widely distributed over the 12 rice chromosomes (Fig. 2). Some genomic regions of < 0.5 Mb containing clusters of M-QTLs affecting multiple related traits were noted, including an M-QTL cluster affecting FLL, FLW and FLA in HF in the 1.2–1.3 Mb interval of chromosome 11. Of the 639 M-QTLs identified, 226 M-QTLs were detected in HF, 204 M-QTLs in NN and 209 M-QTLs detected in DY, with only 28 M-QTLs detected in two of the three environments and 2 M-QTLs (*TGNP1q24* and *FGNP1q25*) detected in all three environments (Fig. 3a). The results suggest that large effects of the three environments and G × E interaction on the trait performances of the RILs and BCF_1_s were primarily reflected by the differential expression (detectability) of M-QTLs.

Based on the ratios of their dominance effects over their additive effects, the identified M-QTLs could be roughly classified into five major types. Of the 639 M-QTLs detected, 273 M-QTLs were of additive gene action, which were more important for HD, PH, TGW, FLL, FLW, PL and PN; 159 M-QTLs showed OD gene action and were more important for GYP, FGNP and SF; 142 M-QTLs exhibited UD gene action and were relatively more important GYP, PN, FGNP and SF; 41 M-QTLs showed CID gene action, most of which were associated with TGW and detected primarily in DY; and only 24 M-QTLs of IR gene action (Fig. 3b–c).

In addition, the importance of epistasis underlying the trait values and heterosis in the QYSM was accessed by inclusive composite interval mapping of the digenic epistatic QTL (E-QTL) across the whole genome. A total of 2736 E-QTL pairs were identified in the combined population. Of these, 38 pairs occurred between one M-QTL and a “background” locus except for one pair between two M-QTLs (*FLA1q15* vs. *FLA9q130*) affecting FLA (Additional file 7: Table S4). When classified according to their gene actions, these E-QTL pairs could be also be classified into 13 major types (Additional file 8: Table S5), including 1199 (43.8%) epistatic gene pairs as OD by UD or UD by OD, 699 (25.5%) OD by OD, 477 (17.4%) UD by UD, with the remaining 13.3% as DA or AD gene actions.

### Distribution of Introgressed *Geng* Alleles in the Q9311B and WSSM Genomes

To determine what portions of the parental (Q9311B and WSSM) genomes were derived from *Xian-Geng* subspecific introgression, we compared Q9311B and WSSM with 1946 diverse rice accessions (772 *Gengs/japonicas* and 1174 *Xians/indicas*) of 3000 rice genomes (3 K-RG) (Wang et al. 2018) for their genomic constitutions (Additional file 9: Fig. S4a). The principal component analysis clearly classified the parents as *Xian* accessions, with Q9311B genetically close to subpopulation *Xian-1A* and WSSM as an admixture between subpopulations *Xian-2* and *Xian-3* (Additional file 9: Fig. S4b). A neighbour-joining tree was developed using 12,139 LD-pruned SNPs with 100 bootstrap replicates revealed that Q9311B and WSSM were both grouped into the same clade *Xian-1B* (Additional file 9: Fig. S4c). By comparing the parental alleles (genic haplotypes) at all 14,397 segregating genes with the gene CDS haplotype database of 3 K-RG (Zhang et al. 2021b), a parental allele at any gene was considered as the *Geng* allele when it had a frequency of > 50% in subpopulation *Geng* and < 10% in subpopulation *Xian*. As a result, Q9311B carries the *Geng* alleles at 1098 genes (7.6%) widely distributed in its genome, while WSSM carries the *Geng* alleles at 305 genes (2.1%) primarily distributed in 14 genomic regions (Additional file 10: Table S6; Additional file 11: Fig. S5). Furthermore, we found that 892 (63.6%) genes with *Geng* alleles carried by the two parents were co-localized in 107 (16.7%) M-QTLs (17 for HD, 14 for SF, 12 for TGNP, 11 for PL, 11 for TGW, 10 for FLL, 9 for FGNP, 7 for GYP, 7 for FLA, 6 for PH and 3 for FLW) (Fig. 2), suggesting the inter-subspecific introgression may have contributed the yield performance of the hybrid, QYSM.

### Important Candidate Genes in Heterotic M-QTL Regions and Their Contributions to Yield Performances of Superior and Inferior BCF_1_ Hybrids

**Fig. 4:**
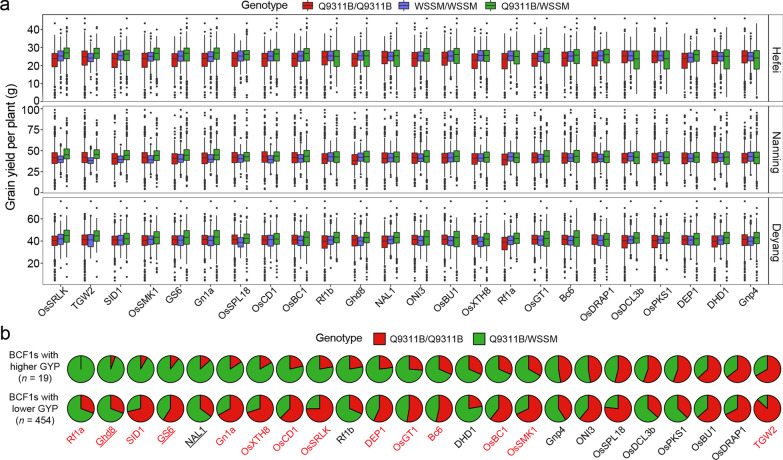
Phenotypic and genotypic differences at 24 cloned genes with heterotic effects on GYP related traits. **a** GYP phenotypic distribution of three genotypes in the RIL and BCF_1_ combined population across three environments. **b** Differences of allelic frequencies between the 19 superior high-yield BCF_1_ hybrids and the 454 infer low-yield BCF_1_ hybrids. Fisher’s exact Test (one-tail) tests were used to determine significant (*P* < 0.05) differences of allelic frequencies between the 19 high-yield BCF_1_ hybrids and the 454 infer low-yield BCF_1_ hybrids. Gene symbols in red indicate significant differences, whereas gene symbols in black indicate no significant difference. Gene symbol with underline represents the allele of one parent may come from *japonica*/*Geng*

To look for possible candidate genes responsible for some of the important heterotic loci, we selected 45 cloned genes co-localized with the identified M-QTLs with strong additive and/or heterotic effects on the same traits measured in this study (Fig. 2; Additional file 6: Table S3 and Additional file 12: Table S7). Of them, each of the cloned genes was found to affect the same suit of traits as its containing M-QTL/genes in at least one of the three environments (Additional file 12: Table S7). More interestingly, we found that the same genes may have different gene actions when affecting different traits, or on the same traits across different environments. For example, M-QTLs (*GYP1q24*, *TGNP1q24* and *FGNP1q23*) containing *OsSRLK* showed OD effect on GYP but with additive effects on TGNP and FGNP in HF, while *TGNP1q24* also exhibited OD effect on TGNP in DY. In addition, *NAL1*, *Ghd8*, *GS6*, *TGW2* and *Gn1a* were found to show significant heterotic effects on the measured yield traits that were reported previously. Gene clusters/M-QTLs containing *NAL1*, *Ghd8*, *GS6*, *TGW2* and *Gn1a* also showed consistent effects on the same suits of traits with their previously reported ones in the gene cloning experiments. Indeed, we observed positive OD for GYP at each of the five genes where the heterozygote showed significantly higher GYP than either of the homozygotes (Fig. 4a). Interestingly, one of the parents (Q9311B and WSSM) carried the *Geng* alleles at *NAL1*, *Ghd8* and *GS6* (Additional file 10: Table S6), suggesting that *Xian*-*Geng* intra-allelic interaction at the three loci might have contributed to the positive heterosis of BCF_1_ hybrids, though their contributions to GYP heterosis through different yield components. At *TGW2* and *Gn1a*, the significant heterotic effects on GYP of the heterozygote were attributable to their strong positive effects for increased TGNP, FGNP, TGW, PL and PH, while the heterotic effect on GYP of *NAL1* was attributable to increased TGNP. Similarly, the positive heterotic effect of *Ghd8* on yield was apparently achieved by its strong effects for increased biomass, TGW and TGNP, while the heterotic effect of *GS6* on increased yield was achieved by increased TGW, TGNP, PL and PH accompanied by delayed HD. However, *OsDCL3b* had strong heterotic effects on significantly increased TGW and PL and delayed HD but accompanied with significantly reduced FGNP, SF, PN and FLL, resulting in a net reduction in GYP. This appeared to be consistent with the observation that the WSSM allele at *OsDCL3b* has its low frequency in population *Xian*. Thus, it suggested that each of the identified gene clusters/M-QTLs contained one or more functional genes contributing to the yield performances of QYSM and its related rice hybrids in China.

To obtain additional evidence that the above candidate genes, gene clusters and important heterotic M-QTLs contributed to the yield performances of BCF_1_ hybrids, we compared 19 superior and 454 inferior BCF_1_ hybrids that showed significantly higher and lower GYP than QYSM in one of the three environments. Figure 4b also showed the allelic frequencies of the 24 heterotic genes in the 19 superior BCF_1_ hybrids and 454 inferior BCF_1_ hybrids, including nine genes (*Rf1a*, *GS6*, *Gn1a*, *OsSRLK*, *Rf1b*, *OsGT1*, *OsSMK1*, *ONI3* and *TGW2*) of positive heterotic effects and eight genes (*OsCD1*, *DEP1*, *OsBC1*, *Gnp4*, *OsSPL18*, *OsDCL3b*, *OsPKS1* and *OsDRAP1*) of UD effects on GYP in the combined population. Compared to the 454 inferior BCF_1_ hybrids, the 19 superior BCF_1_ hybrids showed significantly higher heterozygote frequencies at 14 (*Rf1a*, *Ghd8*, *SID1*, *GS6*, *Gn1a*, *OsXTH8*, *OsCD1*, *OsSRLK*, *DEP1*, *OsGT1*, *Bc6*, *OsBC1*, *OsSMK1* and *TGW2*) of these genes, in which 7 (78%) of 9 genes had positive heterotic effects and 3 (38%) of 8 genes had UD effects. However, none of these genes was observed with significantly lower heterozygote frequencies in the 19 superior BCF_1_ hybrids than the 454 inferior BCF_1_ hybrids. Taking together, it was apparent that individual genes (gene clusters)/QTLs showed varied actions on different yield traits across different environments, and the relatively low level of heterosis at the phenotypic level of QYSM resulted primarily from the larger portion of genes/gene clusters and QTLs of additive and UD gene actions in its parents.

## Discussion

The exploitation of heterosis by developing highly productive hybrids has been demonstrated to be the most successful breeding strategy to increase the productivities of many crops, particularly for open-pollinated crop plants. However, the actual genetic gain for further improving hybrid rice productivity has been slow for several decades despite tremendous breeding efforts. In fact, the two-line rice hybrids have been dominating compared with traditional three-line hybrids in China for more than two decades, which was attributed primarily to its simplicity in hybrid seed production (including the wide presence of restorer lines) and partially to their high yield potential and yield stability from male parents most of which were elite inbred varieties. Thus, dissecting the genetic mechanisms underlying the yield and heterosis of QYSM is expected to help answer the important and puzzling question “what were responsible for the slow genetic gain in the hybrid rice breeding of China?”, at least part of the answer to this question.

### Gene Number and Actions

Three important results were obtained in this regard. First, the number of loci controlling the yield performances and heterosis of yield traits in the Q9311B × WASM RILs and their BCF_1_ hybrids was large, evidenced by the identification of large numbers of segregating genes associated with the yield traits and by the identification of 324 M-QTLs and 1401 E-QTLs affecting GYP and its components (FGNP, PN, TGW, TGNP and SF). The primary reason for the increased power in detecting QTLs/genes was the large population size, multiple environments and high density of markers used in this study. Secondly, compared with the previous reports that loci of overdominance and dominance are the major genetic bases of heterosis of the Lemont/Teqing subspecific BCF_1_/testcross F_1_ hybrid populations and hybrid Shanyou63 (Li et al. 2001; Hua et al. 2003; Zhou et al. 2012), the number and proportion of detected QTLs/gene clusters of additive and UD effects on GYP and its components in hybrid QYSM were much greater. In other words, unexpected higher positon of UD loci affecting yield comparing with previous studies of the similar type. This is not surprising since both Q9311A/B and WSSM were elite varieties with high yield potential and wide adaptability and thus were intensively selected for genes of additive gene actions (Li et al. 2016; Yu et al. 2020). Thus, the presence of large numbers of genes with additive and UD gene actions in the parents of hybrid QYSM was consistent with the fact that QYSM had a relatively low level of heterosis and almost reached the maximum genetic potential of its parents for productivity (few BCF_1_ hybrids outyielded QYSM in each of the environments). This also appeared to provide an adequate explanation for the slow genetic gains of many hybrid rice breeding programs in China. Then, it remains unclear why there are so many UD M-QTLs and gene clusters of negative heterotic effects for GYP and its components in the genomes of the superior parents, Q9311A/B and WSSM. Thirdly, we found little overlap between the QTLs affecting the same traits in the RIL population and those underlying heterosis detected in the BCF_1_ population under the same environments (**Additional file ****5****: **Fig. S3), indicating that genes/QTLs of non-additive gene action mainly contributed the heterosis in rice hybrid while those of additive gene actions largely determined the phenotypic performance of the hybrid parents. In other words, we may improve performance of the hybrid by fixing the favorable alleles of these QTLs of additive gene action in both parents.

### Epistasis and QTL/gene ×  Environment Interaction

Consistent with previous studies (Li et al. 2001, 1997; Yu et al. 1997; Hua et al. 2003), one of the key findings in this study was the importance of epistasis in determining the yield performances and heterosis of QYSM, evidenced by the identification of 1401 E-QTLs affecting GYP and its components occurred mainly between the “background” loci (non-M-QTLs). Moreover, we found that most M-QTLs affecting the yield traits and their heterosis of the BCF_1_ hybrids were largely environment-specific, consistent with previous reports (Zhang et al. 2013; Li et al. 2003; Wang et al. 2014). This also implies that the yield performances and heterosis of the hybrid cultivar, QYSM in different environments were determined by different sets of environmental specific genes/QTLs. Taking together, our results led us to a conclusion that large numbers of genes of different gene actions were involved in the determination of the yield performance and heterosis of rice largely in a trait- and environment specific way and the level of heterosis of a specific rice hybrid in a specific environment is determined jointly by the average effect of all segregating genes of different gene actions that are expressed in the environment.

### Implications to Improving Hybrid Rice

As discussed above, our results that the parents of QYSM carry a large proportion of additive and UD loci was consistent with the relatively low level of heterosis of QYSM. This would be true for most parents of their related hybrids and probably for most parents used in two-line hybrid rice breeding programs in China. This was also consistent with the fact that the yield levels of recently developed elite inbred varieties are approaching that of hybrids in China. Thus, our results have important implications to achieve the accelerated genetic gains in future rice improvement. First, future focus in rice improvement in China should be gradually shifted back to developing high yielding inbred varieties with superior grain quality and resistance/tolerance to multiple biotic and abiotic stresses, particularly in *Xian* rice improvement. Secondly, to break the yield ceiling of hybrid rice in future hybrid rice breeding, increased productivity of hybrids can be achieved by one or combinations of the following three strategies: (1) by pyramiding favorable alleles of genes of additive gene action monomorphic between the parents, (2) by eliminating or minimizing UD loci polymorphic between in the parents, and (3) by pyramiding OD/D genes polymorphic between the parents. Of cause, genomics-based breeding technologies such as genome selection (Cui et al. 2020) and selective introgression (Zhang et al. 2021a) based on accurate genetic information of parental lines and relevant breeding populations are required to realize the proposed strategies. Also, because rice traits differ significantly in their underlying genes of different actions, different breeding strategies should be taken to improve specific trait combinations. For example, TGW, PL, and FLA are largely determined by additive genes, and thus these traits should be more easily improved by phenotypic selection in inbreds. Similarly, TGNP, FGNP and GYP were largely controlled by OD/D loci, suggesting that hybrids are expected to be able to achieve greater values of their maximum TGNP, FGNP and GYP than inbreeds, consistent with the empirical observations. Interestingly, none of the 24 cloned genes with heterotic effects on GYP-related traits had significantly lower heterozygote frequencies in the 19 superior BCF_1_ hybrids than the 454 inferior BCF_1_ hybrids (Fig. 4b). Furthermore, there were significantly higher frequencies at 7 (*Rf1a*, *GS6*, *Gn1a*, *OsSRLK*, *OsGT1*, *OsSMK1* and *TGW2*) of 9 heterotic genes with positive heterotic effects and 3 (*OsCD1*, *DEP1* and *OsBC1*) of 8 heterotic genes with UD effects in the superior BCF_1_ hybrids compared to the inferior BCF_1_ hybrids. These findings strongly suggest the possibility of improving QYSM by pyramiding genes and M-QTLs of positive heterotic effects and by removing genes and M-QTLs of negative heterotic effects.

Clearly, the number of involved genes, their gene actions and affected traits, their interactions with one another and with environments are critical information required for improving complex traits in future genome-based efforts of breeding by design (Zhang et al. 2021a). It should be pointed out that genes with different gene actions appeared to be different from one another is held true only to the specific parents and environments involved. Given the presence of many functional alleles at each of gene loci in the rice germplasm collections (Zhang et al. 2021b), it would be of great interest to know if different intra-allelic combinations at each of the gene loci vary in their gene actions, which will be a major challenge to be addressed in future rice functional genomic research.

It has been proposed to exploit the *Xian*-*Geng* inter-subspecific heterosis as an important strategy to break the yield ceiling of hybrid rice in China because most *Xian*-*Geng* F_1_ hybrids are known to show high levels of heterosis in growth vigor, biomass and sink size except that they tend to show a varied degree of hybrid sterility (Chin et al. 2011; Chu et al. 2012; Dan et al. 2014; Li et al. 1997). To overcome this problem, it has been proposed to develop inter-subspecific hybrids with stronger heterosis and normal seed set by introgressing *Geng* genomes and the wide-compatibility gene (*S5*^*n*^) into *Xian* restorer backgrounds by backcross breeding (Xin et al. 2011; Kim et al. 2017, 2019; Thalapati et al. 2014). This strategy has recently resulted in successful development and releases of several super inter-subspecific hybrids such as Yongyou6, Yongyou9, Yongyou12, Chunyou658, Chunyou84, and Chunyou927 in China (Ma et al. 2007; Lin et al. 2016). These inter-subspecific hybrids showed greater levels of heterosis with maximum yields reaching ~ 15 t/ha (Li et al. 2008, Wang et al. 2013, Li et al. 2016). Nevertheless, it remains critical to determine how much and what specific introgressed *Geng* genome segments (and genes) are responsible for the observed inter-subspecific heterosis. Recently, Lin et al. (Lin et al. 2020) reported a higher level of exogenous genome introgression in hybrid female parents (an average of 22.6%) compared to male parents (an average of 5.74%), which shapes heterotic loci in hybrid rice. In this study, we found that the female parent, Q9311A/B also carries a small but relatively high portion (7.6%) of alleles introgressed from subspecies *Geng* in comparison with male parent WSSM (2.1%), and 63.6% of these subspecific heterozygotes may have contributed to yield performances of the BCF_1_ hybrids varied considerably in both direction and magnitude of their effects on GYP related traits (Additional file 10**: **Table S6 and Additional file 11: Fig. S5). For example, *NAL1* and *Ghd8* with *Geng* alleles in WSSM and *GS6* with *Geng* allele in Q9311A/B (Fig. 4b) might have contributed to the positive heterosis in hybrid QYSM through different yield components, which is consistent with the findings of Lin et al. (Lin et al., 2020). In addition, we have recently found that ~ 20,000 of the rice genes are *Xian-Geng* differentiating genes at each of which there are two predominant alleles, one in subspecies *Xian* and the other in *Geng* (Zhang et al. 2021b). Thus, what proportion and how this group of *Xian-Geng* differentiating genes contribute to the inter-subspecific heterosis and *Xian*-*Geng* hybrid sterility would be the key for full exploitation of the *Xian*-*Geng* heterosis in future hybrid rice breeding.

## Conclusions

Through deep genome sequencing, extensive phenotyping of the very large RIL and BCF_1_ hybrid populations across three environments, and accurate resolution of genome-wide segregating genes of different types of gene actions on the measured traits, we were able to identify large numbers of QTLs/genes of significant additive and non-additive effects on 12 rice yield-related traits. Our results suggest that hybrid productivity can be increased by one or combinations of the three strategies: (1) by pyramiding favorable alleles of additive genes, (2) by eliminating or minimizing UD loci, and (3) by pyramiding OD/D genes polymorphic, particularly those underlying *Xian*-*Geng* inter-subspecific heterosis.

## Methods

### Experimental Materials

Two populations used in this study included a set of 1061 F_2:8_ RILs derived from the single-seed descent from a cross between an elite *Xian* maintainer line, Quan9311B (Q9311B) and an elite *Xian* restorer line, Wu-shan-si-miao (WSSM) and a BCF_1_ population developed by crossing the RILs (used as male) to the cytoplasmic male sterile (CMS) line Quan9311A (Q9311A). Q9311A is a stable *Xian* CMS line with excellent general combining ability and has been extensively used as a backbone line in the three-line CMS hybrid rice breeding programs in China. In addition, the parents Q9311B and WSSM, and the F_1_ hybrid (Q9311A × WSSM, abbreviated as QYSM) were used as the checks in the phenotyping experiments.

### Field Experiment and Trait Evaluation

Field experiments were conducted at three experimental stations of the Win-All Hi-Tech Seed Co., Ltd in three different locations, including Hefei (HF, 31° N, 117° E) of Anhui Province, Nanning (NN, 22° N, 108° E) of Guangxi Province and Deyang (DY, 31° N, 104° E) of Sichuan Province. The RILs, BCF_1_s, F_1_ hybrid QYSM and their parents (Q9311B and WSSM) were sowed in the seedling nursery on May 17 2019, July 12 2019, and April 22 2019 in the HF, NN, and DY experiment, respectively. The 25-day-old seedlings of RILs and BCF_1_s were transplanted into four-row plots with two replications by a randomized complete block design. Each row within a plot consisted of 6 plants with a spacing of 17 cm between the plants and 20 cm between rows. Three check plots consisting of Q9311B, WSSM, and their F_1_ hybrid QYSM were randomly arranged in each replication. Crop management followed local field production practices in the three locations. Twelve agronomic traits (heading date (HD), plant height (PH), flag leaf length (FLL), flag leaf width (FLW), flag leaf area (FLA), panicle length (PL), panicle number per plant (PN), total grain number per panicle (TGNP), filled grain number per panicle (FGNP), spikelet fertility (SF), 1000-grain weight (TGW), grain yield per plant (GYP)) were evaluated. Measurements for the FLW (mm) and FLL (cm) were taken on 2 main stems of each plant in the middle 3 plants of each plot. FLA was calculated by the formula of FLL × FLW × 0.75. At the maturity, PN was investigated from the middle 3 plants each plot. TGNP and FGNP were measured from 4 main panicles from the middle 4 plants (one main panicle each plant) each plot. SF was calculated by the ratio FGNP to TGNP. An estimate of the TGW (g) was calculated by dividing the filled grain weight by the filled grain number of the 4 main panicles and then multiplying 1000. The grain yield each plot was obtained after weighing all grains collected from the rest panicles and the 4 main panicles of each plot. Then GYP (g) was calculated by the ratio of total grain yield to the number of plants in each plot.

### Whole-Genome Sequencing of the Parents and RILs

The parents, Q9311B and WSSM, and the 1061 RILs were sequenced using the NGS platform. Genomic DNA for SNP genotyping was isolated from approximately 100 mg fresh leaf samples of 5-week-old seedlings for each of the 1061 RILs, Q9311B and WSSM using a modified cetyltrimethylammonium bromide method (Murray and Thompson 1980). DNA degradation and contamination were monitored on 0.8% agarose gels. DNA purity was checked using the NanoPhotometer® spectrophotometer (IMPLEN, CA, USA). DNA concentration was measured using Qubit® DNA Assay Kit in Qubit^®^ 3.0 Fluorometer (Life Technologies, CA, USA).

The NEB Next^®^ Ultra DNA Library Prep Kit for Illumina^®^ (NEB, USA) was used to construct the libraries for sequencing as per the manufacturer’s instructions. DNA was fragmented into ~ 200 base pair pieces. The end of the DNA fragment was subjected to an end repair process that included the addition of a single “A” base, followed by ligation of the adapters. Products were purified and enriched by polymerase chain reaction (PCR) to amplify the library DNA. The final libraries were quantified using KAPA Library Quantification kit (KAPA Biosystems, South Africa) and an Agilent 2100 Bioanalyzer. Paired-end sequencing (2 × 150 basepair) was performed on an Illumina NovaSeq 6000 sequencer (Illumina, USA).

### SNP Calling, Gene-Based Allele Genotyping and Bin-Map Construction

Overall, sequencing of the parental lines (Q9311B and WSSM) and 1061 RILs generated 150 bp paired-end reads containing totally ~ 2.4 Tb sequences. Each parental line had approximately 40 × genome coverage, and each RIL had approximately 10 × genome coverage. The sequence reads of the parental lines and all RILs were aligned against the Nipponbare reference genome (IRGSP 1.0) (Kawahara et al. 2013) with the BWA package (version 0.7.1) using default parameters, and PCR duplicates were removed by the ‘MarkDuplicates’ module in the Picard tools (version 1.119) (Li and Durbin 2009). The raw reads were also re-aligned for highly polymorphic regions using the ‘IndelRealigner’ function in GenomeAnalysisTK (version 3.4.0) (Danecek et al. 2011). The sequence variants between the parental lines were called using ‘UnifiedGenotyper’ in the GenomeAnalysisTK. Only the uniquely mapped reads were used for subsequent SNP calling. Genotype calling of each RIL was carried out based on the SNP alleles between the parents. A total of 741,928 homozygous SNPs polymorphic between Q9311B and WSSM were used for SNP calling in the 1061 RILs. Furthermore, we filtered the SNPs by removing those with minor allele frequencies < 0.01 or missing rates > 0.2, leaving 156,373 high-quality SNPs for further gene-based allelic genotyping.

The 156,373 SNPs locate within the genic regions of 15,043 genes based on the gene annotation of the Nipponbare RefSeq from RAP-DB (released on June 26, 2019) (Sakai et al. 2013). Allelic genotypes (Q9311B type, WSSM type, and the heterozygote) of the 15,043 genes were constructed by concatenating the SNPs in each of their genic regions. We removed 1196 genes because one of the two parental alleles was rare or had a very high frequency of the heterozygote in the RIL population. Finally, an allelic genotyping dataset containing three genotypes at each of 13,847 genes was generated for the 1061 RILs to be used in further data analyses. The genotypes for each BCF_1_ hybrid were deduced from the gene-based allelic genotypes of its parental RIL and Q9311B. Specifically, for each of the 13,847 genes, if the parents (one RIL and Q9311B) have the same allelic genotype, their BCF_1_ hybrid should be the homozygote genotype of Q9311B; when the parents have different homozygous genotypes, the allelic genotype of their BCF_1_ hybrid was deduced as the heterozygote; and when for those BCF_1_ hybrids with a heterozygous RIL parent, these BCF_1_s were treated as missing.

The gene-based allelic genotypic data of 13,847 genes of the RIL population were used for bin map construction using the BIN function in IciMapping QTL version 4.2 (Meng et al. 2015). To remove redundancy, only one gene was retained to represent each bin, either one gene with a minimum missing rate, or a random gene when the missing rate was equal. The genes which displayed a unique pattern of segregation and did not fall into a bin were removed. As a result, 855 bins were identified and used for constructing the genetic linkage map of the RIL population with the recombination frequencies and Kosambi mapping function with IciMapping QTL version 4.2 (Meng et al. 2015). The constructed linkage map spanned a total length of 2268 cM with an average of 2.69 cM between neighbouring bins.

### QTL Mapping

QTL mapping for 12 yield-related traits was performed separately for the RIL population, BCF_1_ hybrid population, and a combined population containing all RILs and BCF_1_s evaluated in each of the environments. To minimize the possible influence of abnormal fertility on yield-related traits in the BCF_1_s resulting from absent restoring gene in RILs, we removed 152 BCF_1_s with the low (< 50%) spikelet fertility in all of the three locations for further data analyse. For each population, the mean trait values from two replications at each environment were used as input data. For the BCF_1_ hybrid population, the mean trait values and mid-parental heterosis values (*H*_MP_), *H*_MP_ = [(BCF_1 _− (P_1_ + P_2_)/2)/(P_1_ + P_2_)/2)] × 100, were used as additional input data, where P_1_ and P_2_ were the mean trait values of Q9311B and the parental RIL. Then, all three populations were analyzed using the BIP (biparental populations) function in IciMapping QTL version 4.2 (Meng et al. 2015). The parameter of mapping population type for the combined population was set as “BC1F2” because both its expected and observed genotype frequencies fit the segregation ratio of BC_1_F_2_ population (Additional file 4: Fig. S2; Additional file 13: Table S8). The inclusive composite interval mapping of additive (ICIM-ADD) QTL method was performed to identify main-effect QTLs (M-QTLs) using the default settings. The analyses of M-QTLs were performed with pre-adjusted IciMapping parameters, in which *P* values for entering a variable (PIN) were set at 0.001 and the scanning step was set at 1.0 cM. The inclusive composite interval mapping of the digenic epistatic (ICIM-EPI) QTL method was used to detect possible digenic epistatic QTLs (E-QTLs) using the default settings. The corresponding scan step and PIN for E-QTLs mapping were set at 5 cM and 0.0001, respectively. The LOD threshold for M-QTLs was 3.7 determined by a permutation test with 1000 runs, and the LOD threshold 5.0 was used to declare significant E-QTLs. The physical position of each detected QTL was retrieved based on the left and right markers of the detected interval. All known genes underlying related traits within an identified QTL interval were considered as candidate genes based on the Nipponbare reference genome (IRGSP 1.0)(Kawahara et al. 2013). All identified QTLs were named as “trait abbreviation + chromosome number + q + position”.

### Inferences of QTL Gene Actions

Gene action of each of the detected M-QTLs was inferred according to the ratio of its estimated dominance effect (*d*) to its estimated additive effect (*a*) according to the Liu et al.’s method (Liu et al. 2020). Briefly, the parameters, *a* and *d* were estimated from the mean trait data of the combined population containing all RILs and BCF_1_s. Statistical tests were performed through the analysis of variance (ANOVA), to determine the significance levels of the heterotic effects (the estimates of *d* and *a* values) of all identified M-QTLs. Then, QTL gene actions could roughly be divided into five types: overdominance (OD, *d*/|*a*|> 1.25), complete-incomplete dominance (CID, 0.25 < *d*/|*a*|≤ 1.25), additive (− 0.25 < *d*/|a|≤ 0.25), incomplete recessiveness (IR, − 1.25 < *d*/|*a*|≤ − 0.25) and underdominance (UD, *d*/|*a*|≤ − 1.25). The average phenotypic measurements of heterozygous and homozygous genotypes were further calculated for estimating dominance effect index (*d*/*a*) and *H*_MP_ index for each M-QTL.

### Determination of Introgressed *Geng* Alleles in the Q9311B and WSSM Genomes

The frequencies of the Q9311B and WSSM alleles in subspecies *Xian* and *Geng* were calculated for 12,722 of the 13,847 genes polymorphic between the parents. Specifically, we extracted a subset of SNP data for 1946 accessions (1174 *Xians* and 772 *Gengs*) from 4.8 M filtered SNP Dataset of 3 K-RG (Mansueto et al. 2016), and merged the extracted SNPs with the polymorphic SNPs between Q9311B and WSSM, resulting in 624,049 overlapping SNPs in PLINK (Purcell et al. 2007). Of the 624,049 SNPs, 133,587 SNPs are located within the genic regions of 14,397 genes based on the gene annotation of Nipponbare RefSeq from RAP-DB (released on June 26, 2019) (Sakai et al. 2013). Finally, a genic haplotype dataset containing 14,397 genes was generated by concatenating the SNPs across the genomes of the 1946 accessions from 3 K-RG and the parents (Q9311B and WSSM). The haplotype frequencies of Q9311B and WSSM at each gene in the 1946 accessions were calculated to infer the nature of the Q9311B and WSSM alleles. When the Q9311B or WSSM allele at a gene is present in > 50% in population *Geng* and < 10% in population *Xian*, it was considered as a *Geng* allele.

### Statistical Analysis

Analyses of variance were performed to determine significant variation between locations and genotypes for all measured traits by the agricolae package in R. Significant phenotypic differences among the check parents and the relative hybrids using Duncan’s multiple comparison test, and among RILs and the BCF_1_s were statistically assessed using Student’s *t*-test by the agricolae package in R. *H*_MP_ was tested with a Student’s *t*-test based on the contrast between F_1_ hybrid mean and average performance of corresponding parental lines (Larièpe et al. 2012). Pearson’s correlation analyses among the phenotypic traits measured were performed by the Hmisc package in R. One-tailed Fisher’s exact tests were performed with the ‘‘fisher.test’’ function in R to determine significant differences in allelic frequencies.

## Supplementary Information

**Additional file 1: Table S1**. Phenotypic performance and heterosis of traits in Q9311B/WSSM RILs and backcross F_1_s (Q9311A × RIL).

**Additional file 2: Table S2**. Some summary statistics from two-way analysis of variance of 12 traits of rice hybrid QYSM, derived RIL and BCF1 hybrid (RIL × Q9311) populations evaluated in three environments (Hefei, Nanning and Deyang).

**Additional file 3: Fig. S1**. Phenotypic distribution for 12 traits at the three environments. a, In recombined inbred lines (RILs). b, In backcross F1s hybrids (BCF1s). c, Mid-parental heterosis values. d, Over-standard heterosis values. Over-standard heterosis was calculated by [(BCF1 – QYSM)/QYSM] × 100. RILs derived from a cross between Q9311B and WSSM, BCF1s were hybrids between the RILs and Q9311A. HD, heading date; PH, plant height; PN, panicle number per plant; FLL, flag leaf length; FLW, flag leaf width; FLA, flag leaf area; PL, panicle length; FGNP, filled grain number per plant; TGNP, total grain number per plant; SF, spikelet fertility; TGW, 1000-grain weight; GYP, grain yield per plant.

**Additional file 4: Fig. S2**. High-density linkage map consisting of 855 bins was constructed for the RIL population from a cross between Q9311B and WSSM based on the genotypes at 13,847 segregating genes. a, The recombination bin map of the RIL population (*n* = 1061), in which the horizontal axis indicates the RILs and the vertical axis indicates genomic regions. Q9311B/Q9311B homozygous type is shown in red, WSSM/WSSM homozygous type is shown in blue, and Q9311B/WSSM heterozygous type is shown in grey. b, Plots of the Q9311 allele frequency for each bin in the RIL population.

**Additional file 5: Fig. S3**. Genomic distribution of main-effect QTLs affecting 12 rice yield related traits identified in the RIL and BCF_1_ populations separately.

**Additional file 6: Table S3**. Summary of 639 main-effect QTL affecting 12 traits identified in the combined population across three environments by inclusive composite interval mapping.

**Additional file 7: Table S4**. Epistasis QTLs for 12 traits in the three environments.

**Additional file 8: Table S5**. Classification of 2,736 epistatic QTL pairs into 13 types according to their gene actions identified in the Q9311/WSSM combined population.

**Additional file 9: Fig. S4**. Whole-genome variation of two parental lines of QYSM. a, Density and chromosome distribution of polymorphic SNPs between Q9311B and WSSM. b, The principal component analysis. c, Phylogenetic positions of two parental lines (Q9311B and WSSM) in the neighbor-joining tree of 1,946 rice accessions from 3 K-RG, in which the known information on the clades of 3 K-RG is indicated.

**Additional file 10: Table S6**. Allele frequencies of Q9311B and WSSM in 1946 accessions from 3 K-RG.

**Additional file 11: Fig. S5**. Speculation of the subpopulation source of the parental allele at all 14,397 segregating genes by comparing the allele frequencies of Q9311B and WSSM in 772 *Geng* and 1,174 *Xian* accessions from 3 K-RG.

**Additional file 12: Table S7**. The function of 45 cloned yield related genes identified as the candidate genes for determining the performance of hybrid rice variety QYSM.

**Additional file 13: Table S8**. The genotypic and phenotypic matrix of the population in ICIMapping format.

## Data Availability

All data supporting the conclusions of this article are provided within the article (and in the Additional files).

## Abbreviations

CMS: Cytoplasmic male sterility
QTL: Quantitative trait locus
GWAS: Genome-wide association study
SNP: Single nucleotide polymorphism
QYSM: Quan-you-si-miao
GCA: General combining ability
Q9311: Quan9311
WSSM: Wu-shan-si-miao
RIL: Recombined inbred line
HD: Heading date
PH: Plant height
FLL: Flag leaf length
FLW: Flag leaf width
FLA: Flag leaf area
PL: Panicle length
PN: Panicle number per plant
TGNP: Total grain number per panicle
FGNP: Filled grain number per panicle
SF: Spikelet fertility
TGW: 1000-Grain weight
GYP: Grain yield per plant
M-QTL: Main-effect QTL
E-QTL: Epistatic QTL
ANOVA: Analysis of variance
OD: Overdominance
CID: Complete-incomplete dominance
IR: Incomplete recessiveness
UD: Underdominance
PVE: Phenotypic variation explained
HF: Hefei
NN: Nanning
DY: Deyang
