# Supplementary figures and images for: Genomic Architecture of Yield Performance of an Elite Rice Hybrid Revealed by its Derived Recombinant Inbred Line and Their Backcross Hybrid Populations
Source: Rice (N Y). 2022 Oct 1;15:49. doi: 10.1186/s12284-022-00595-z (PMC9526777; doi:10.1186/s12284-022-00595-z)

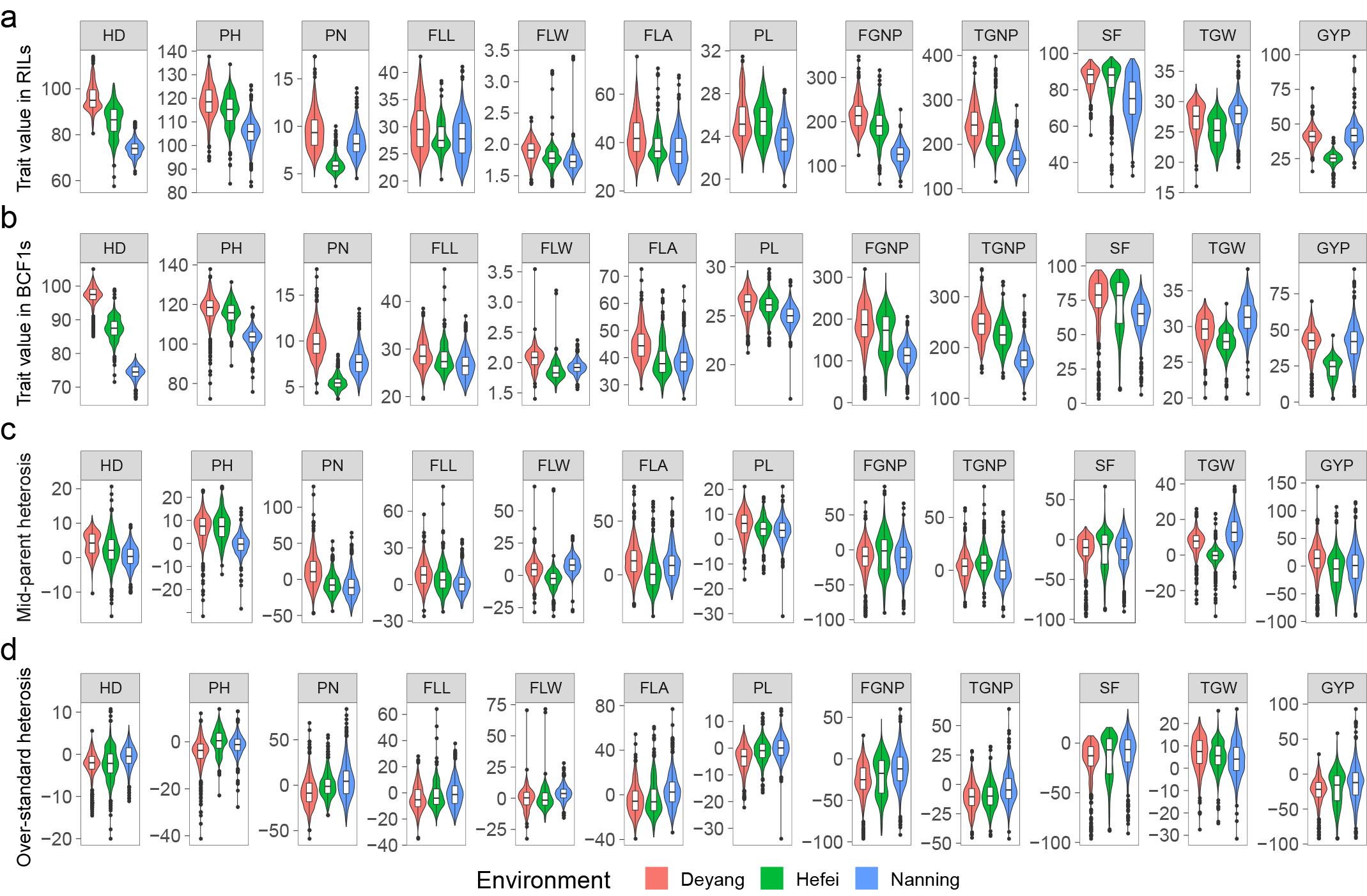

Supplement: Supplementary file 3 — Additional file 3: Fig. S1. Phenotypic distribution for 12 traits at the three environments. a, In recombined inbred lines (RILs). b, In backcross F1s hybrids (BCF1s). c, Mid-parental heterosis values. d, Over-standard heterosis values. Over-standard heterosis was calculated by [(BCF1 – QYSM)/QYSM] × 100. RILs derived from a cross between Q9311B and WSSM, BCF1s were hybrids between the RILs and Q9311A. HD, heading date; PH, plant height; PN, panicle number per plant; FLL, flag leaf length; FLW, flag leaf width; FLA, flag leaf area; PL, panicle length; FGNP, filled grain number per plant; TGNP, total grain number per plant; SF, spikelet fertility; TGW, 1000-grain weight; GYP, grain yield per plant. [file 12284_2022_595_MOESM3_ESM.tif]

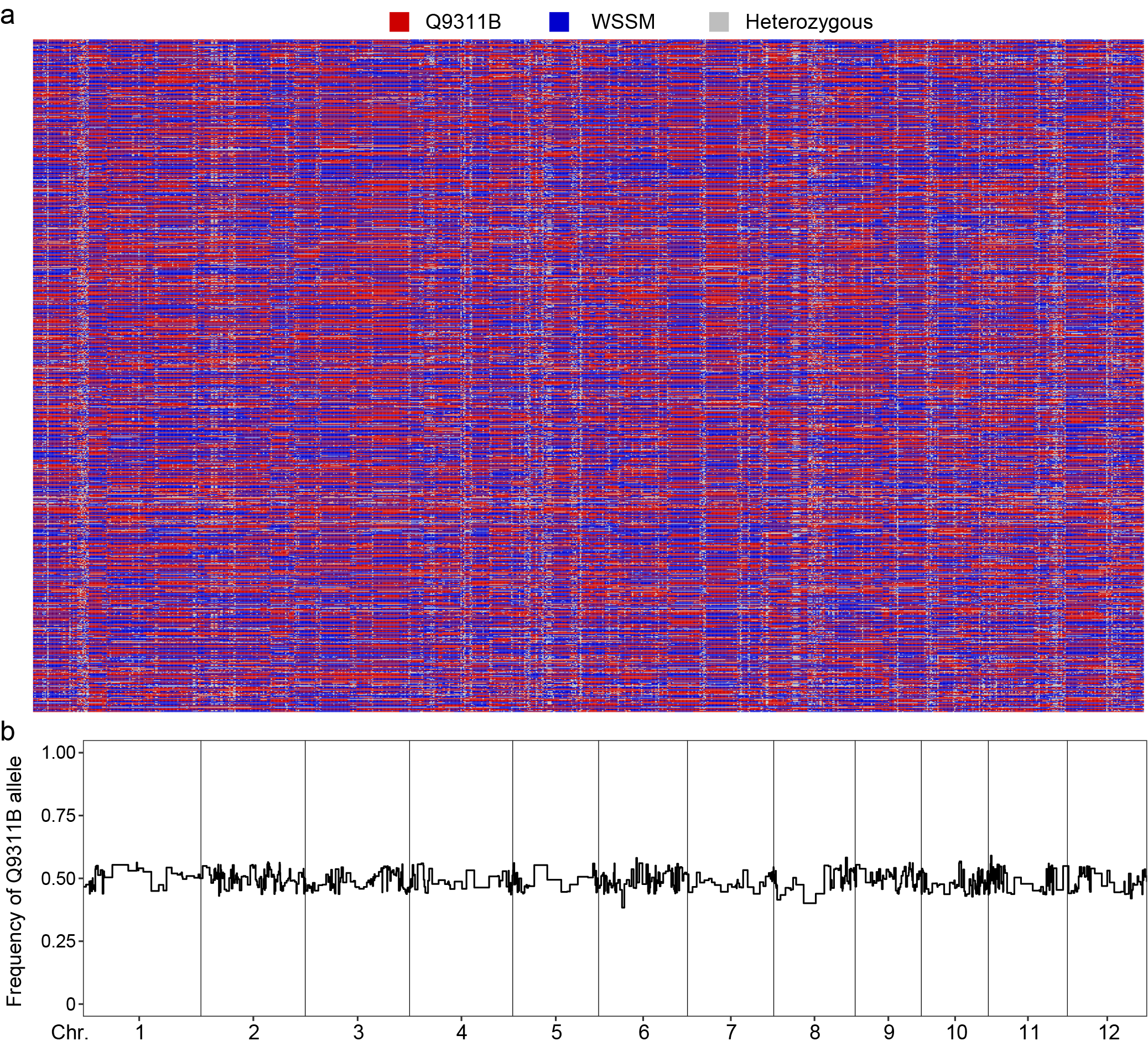

Supplement: Supplementary file 4 — Additional file 4: Fig. S2. High-density linkage map consisting of 855 bins was constructed for the RIL population from a cross between Q9311B and WSSM based on the genotypes at 13,847 segregating genes. a, The recombination bin map of the RIL population (n = 1061), in which the horizontal axis indicates the RILs and the vertical axis indicates genomic regions. Q9311B/Q9311B homozygous type is shown in red, WSSM/WSSM homozygous type is shown in blue, and Q9311B/WSSM heterozygous type is shown in grey. b, Plots of the Q9311 allele frequency for each bin in the RIL population. [file 12284_2022_595_MOESM4_ESM.tif]

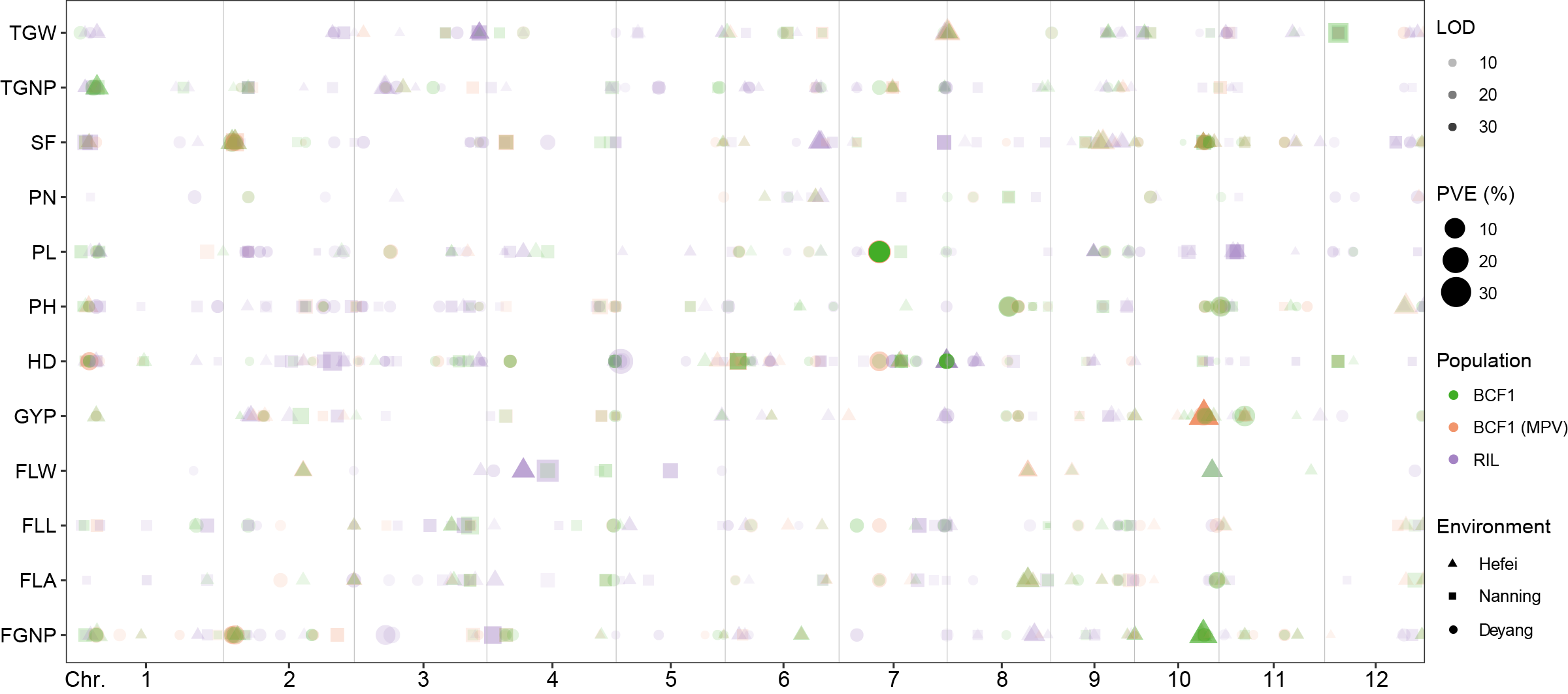

Supplement: Supplementary file 5 — Additional file 5: Fig. S3. Genomic distribution of main-effect QTLs affecting 12 rice yield related traits identified in the RIL and BCF1 populations separately. [file 12284_2022_595_MOESM5_ESM.tif]

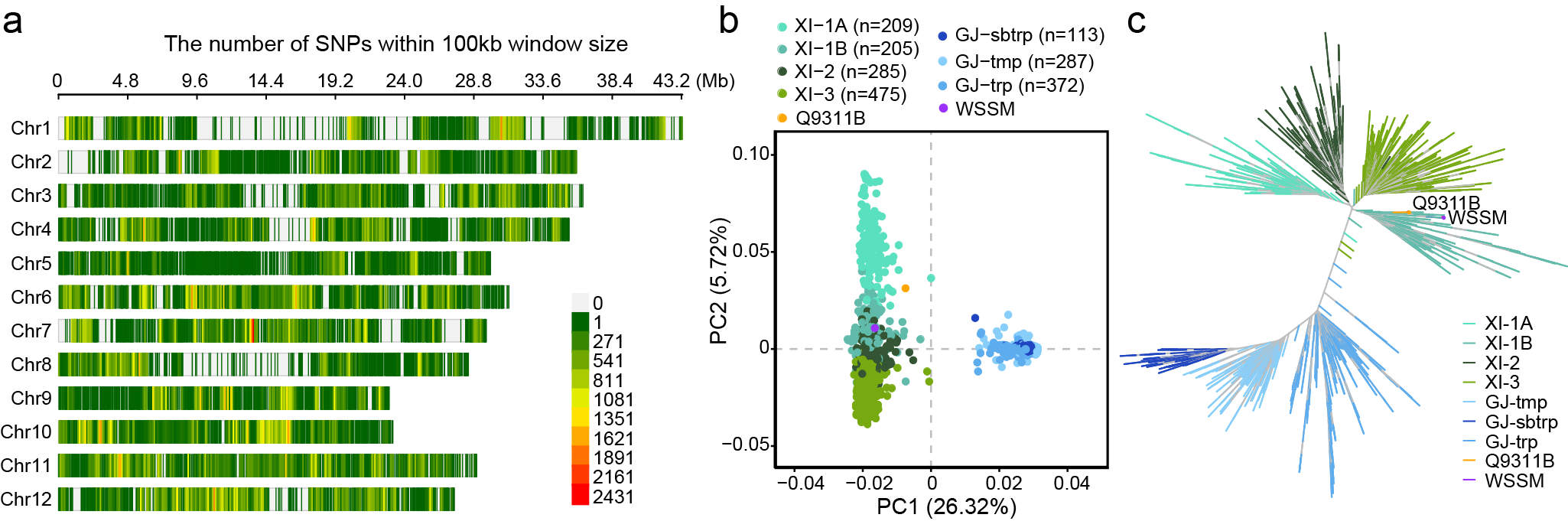

Supplement: Supplementary file 9 — Additional file 9: Fig. S4. Whole-genome variation of two parental lines of QYSM. a, Density and chromosome distribution of polymorphic SNPs between Q9311B and WSSM. b, The principal component analysis. c, Phylogenetic positions of two parental lines (Q9311B and WSSM) in the neighbor-joining tree of 1,946 rice accessions from 3 K-RG, in which the known information on the clades of 3 K-RG is indicated. [file 12284_2022_595_MOESM9_ESM.tif]

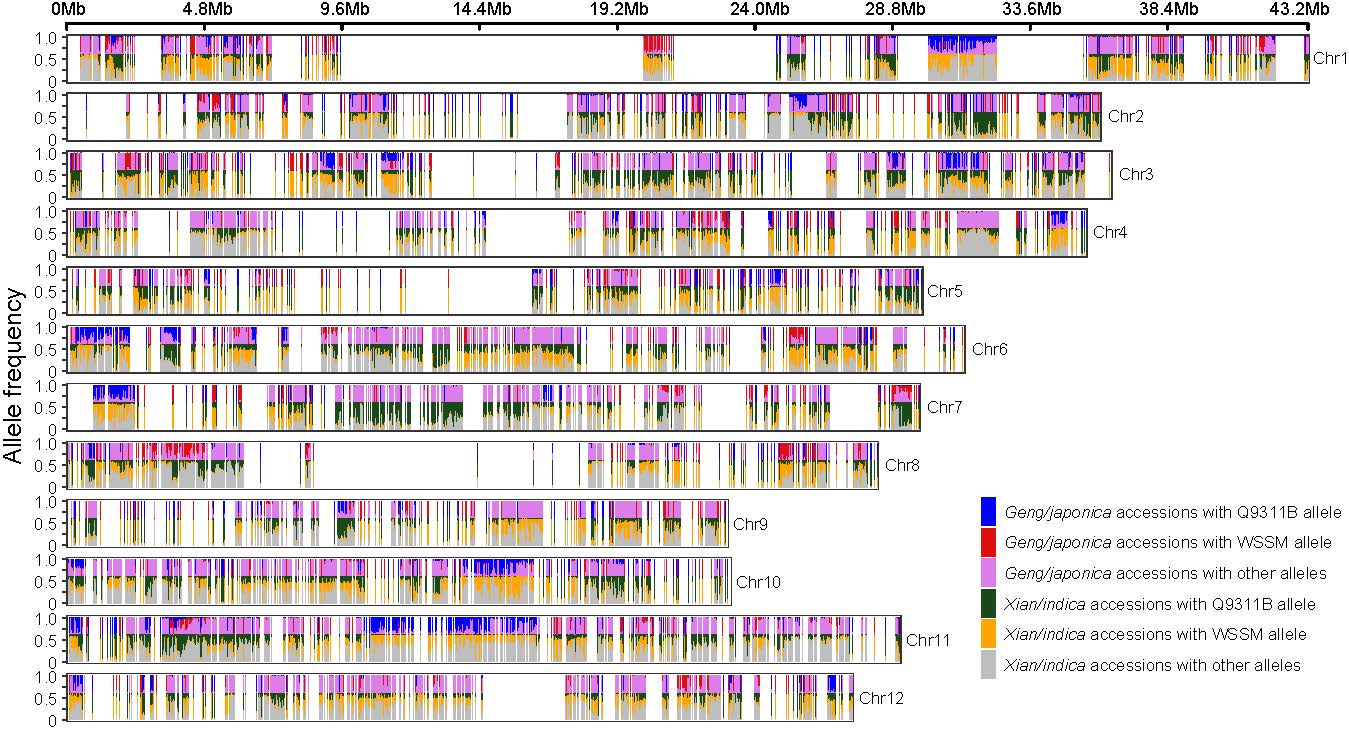

Supplement: Supplementary file 11 — Additional file 11: Fig. S5. Speculation of the subpopulation source of the parental allele at all 14,397 segregating genes by comparing the allele frequencies of Q9311B and WSSM in 772 Geng and 1,174 Xian accessions from 3 K-RG. [file 12284_2022_595_MOESM11_ESM.jpg]
